# A Genome-Wide Association Study Reveals Variants in *ARL15* that Influence Adiponectin Levels

**DOI:** 10.1371/journal.pgen.1000768

**Published:** 2009-12-11

**Authors:** J. Brent Richards, Dawn Waterworth, Stephen O'Rahilly, Marie-France Hivert, Ruth J. F. Loos, John R. B. Perry, Toshiko Tanaka, Nicholas John Timpson, Robert K. Semple, Nicole Soranzo, Kijoung Song, Nuno Rocha, Elin Grundberg, Josée Dupuis, Jose C. Florez, Claudia Langenberg, Inga Prokopenko, Richa Saxena, Robert Sladek, Yurii Aulchenko, David Evans, Gerard Waeber, Jeanette Erdmann, Mary-Susan Burnett, Naveed Sattar, Joseph Devaney, Christina Willenborg, Aroon Hingorani, Jaquelin C. M. Witteman, Peter Vollenweider, Beate Glaser, Christian Hengstenberg, Luigi Ferrucci, David Melzer, Klaus Stark, John Deanfield, Janina Winogradow, Martina Grassl, Alistair S. Hall, Josephine M. Egan, John R. Thompson, Sally L. Ricketts, Inke R. König, Wibke Reinhard, Scott Grundy, H-Erich Wichmann, Phil Barter, Robert Mahley, Y. Antero Kesaniemi, Daniel J. Rader, Muredach P. Reilly, Stephen E. Epstein, Alexandre F. R. Stewart, Cornelia M. Van Duijn, Heribert Schunkert, Keith Burling, Panos Deloukas, Tomi Pastinen, Nilesh J. Samani, Ruth McPherson, George Davey Smith, Timothy M. Frayling, Nicholas J. Wareham, James B. Meigs, Vincent Mooser, Tim D. Spector, GIANT Consortium

**Affiliations:** 1 Departments of Medicine, Human Genetics, and Epidemiology and Biostatistics, Jewish General Hospital, McGill University, Montréal, Québec, Canada; 2 Twin Research and Genetic Epidemiology, King's College London, London, United Kingdom; 3 Genetics Division, GlaxoSmithKline, King of Prussia, Pennsylvania, United States of America; 4 University of Cambridge Metabolic Research Laboratories, Institute of Metabolic Science, Addenbrooke's Hospital, University of Cambridge, Cambridge, United Kingdom; 5 General Medicine Division, Massachusetts General Hospital, Boston, Massachusetts, United States of America; 6 Department of Medicine, Harvard Medical School, Boston, Massachusetts, United States of America; 7 Medical Research Council Epidemiology Unit, Institute of Metabolic Science, Addenbrooke's Hospital, Cambridge, United Kingdom; 8 Genetics of Complex Traits, Institute of Biomedical and Clinical Science, Peninsula Medical School, Exeter, United Kingdom; 9 Diabetes Genetics, Institute of Biomedical and Clinical Science, Peninsula Medical School, Exeter, United Kingdom; 10 Clinical Research Branch, National Institute on Aging, Baltimore, Maryland, United States of America; 11 Medstar Research Institute, Baltimore, Maryland, United States of America; 12 Medical Research Council Centre for Causal Analyses in Translational Epidemiology, Department of Social Medicine, Oakfield House, Bristol, United Kingdom; 13 Wellcome Trust Sanger Institute, Wellcome Trust Genome Campus, Hinxton, United Kingdom; 14 McGill University and Genome Québec Innovation Center, Montréal, Québec, Canada; 15 Department of Biostatistics, School of Public Health, Boston University, Boston, Massachusetts, United States of America; 16 Center for Human, Genetic Research and Diabetes Center (Diabetes Unit), Department of Medicine, Massachusetts General Hospital, Boston, Massachusetts, United States of America; 17 Program in Medical and Population Genetics, Broad Institute of Harvard and Massachusetts Institute of Technology, Cambridge, Massachusetts, United States of America; 18 Oxford Centre for Diabetes, Endocrinology and Metabolism, University of Oxford, Oxford, United Kingdom; Wellcome Trust Centre for Human Genetics, University of Oxford, Oxford, United Kingdom; 19 Department of Epidemiology, Erasmus Medical Center, Rotterdam, the Netherlands; 20 Department of Internal Medicine, Centre Hospitalier Universitaire Vaudois (CHUV) University Hospital, Lausanne, Switzerland; 21 Medizinische Klinik II, Universität zu Lübeck, Lübeck, Germany; 22 Cardiovascular Research Institute, Washington Hospital Center, Washington, District of Columbia, United States of America; 23 British Heart Foundation Glasgow Cardiovascular Research Centre, University of Glasgow, Glasgow, United Kingdom; 24 Institut für Medizinische Biometrie und Statistik, Universität zu Lübeck, Lübeck, Germany; 25 Centre for Clinical Pharmacology, University College, London, United Kingdom; 26 Klinik und Poliklinik für Innere Medizin II, Universität Regensburg, Regensburg, Germany; 27 Cardiothoracic Unit, Great Ormond Street Hospital for Children National Health Service Trust, London, United Kingdom; 28 Leeds Institute of Genetics, Health and Therapeutics and Leeds Institute of Molecular Medicine, Faculty of Medicine and Health, University of Leeds, Leeds, United Kingdom; 29 Laboratory of Clinical Investigation, National Institute of Aging, Baltimore, Maryland, United States of America; 30 Departments of Health Sciences, University of Leicester, Leicester, United Kingdom; 31 Department of Public Health and Primary Care, Strangeways Research Laboratory, University of Cambridge, Cambridge, United Kingdom; 32 Center for Human Nutrition, Department of Clinical Nutrition, University of Texas, Southwestern Medical Center, Dallas, Texas, United States of America; 33 Institute of Epidemiology, Helmholtz Zentrum München, German Research Center for Environmental Health, Neuherberg, Germany; Institute of Medical Information Science, Biometry and Epidemiology, Ludwig-Maximilans-Universität, Munich, Germany; 34 Heart Research Institute, Camperdown, Sydney, New South Wales, Australia; 35 Gladstone Institute of Neurological Disease and Gladstone Institute of Cardiovascular Disease, San Francisco, California, United States of America; 36 Department of Internal Medicine and Biocenter Oulu, University of Oulu, Oulu, Finland; 37 Cardiovascular Institute and Institute for Translational Medicine and Therapeutics, University of Pennsylvania School of Medicine, Philadelphia, Pennsylvania, United States of America; 38 Division of Cardiology, University of Ottawa Heart Institute, Ottawa, Ontario, Canada; 39 Department of Cardiovascular Sciences, University of Leicester, Glenfield Hospital, Leicester, United Kingdom; University of Michigan, United States of America

## Abstract

The adipocyte-derived protein adiponectin is highly heritable and inversely associated with risk of type 2 diabetes mellitus (T2D) and coronary heart disease (CHD). We meta-analyzed 3 genome-wide association studies for circulating adiponectin levels (n = 8,531) and sought validation of the lead single nucleotide polymorphisms (SNPs) in 5 additional cohorts (n = 6,202). Five SNPs were genome-wide significant in their relationship with adiponectin (P≤5×10^−8^). We then tested whether these 5 SNPs were associated with risk of T2D and CHD using a Bonferroni-corrected threshold of P≤0.011 to declare statistical significance for these disease associations. SNPs at the adiponectin-encoding *ADIPOQ* locus demonstrated the strongest associations with adiponectin levels (P-combined = 9.2×10^−19^ for lead SNP, rs266717, n = 14,733). A novel variant in the *ARL15* (ADP-ribosylation factor-like 15) gene was associated with lower circulating levels of adiponectin (rs4311394-G, P-combined = 2.9×10^−8^, n = 14,733). This same risk allele at *ARL15* was also associated with a higher risk of CHD (odds ratio [OR] = 1.12, P = 8.5×10^−6^, n = 22,421) more nominally, an increased risk of T2D (OR = 1.11, P = 3.2×10^−3^, n = 10,128), and several metabolic traits. Expression studies in humans indicated that ARL15 is well-expressed in skeletal muscle. These findings identify a novel protein, ARL15, which influences circulating adiponectin levels and may impact upon CHD risk.

## Author Summary

Through a meta-analysis of genome-wide association studies of 14,733 individuals, we identified common base-pair variants in the genome which influence circulating adiponectin levels. Since adiponectin is an adipocyte-derived circulating protein which has been inversely associated with risk of obesity-related diseases such as type 2 diabetes (T2D) and coronary heart disease (CHD), we next sought to understand if the identified variants influencing adiponectin levels also influence risk of T2D, CHD, and several metabolic traits. In addition to confirming that variation at the *ADIPOQ* locus influences adiponectin levels, our analyses point to a variant in the *ARL15* (ADP-ribosylation factor-like 15) locus which decreases adiponectin levels and increases risk of CHD and T2D. Further, this same variant was associated with increased fasting insulin levels and glycated hemoglobin. While the function of *ARL15* is not known, we provide insight into the tissue specificity of *ARL15* expression. These results thus provide novel insights into the physiology of the adiponectin pathway and obesity-related diseases.

## Introduction

Adiponectin is an adipocyte-secreted protein that increases insulin sensitivity [1],[2],[3], and has anti-diabetic [4],[5],[6] and anti-atherogenic effects [7]. Several features render adiponectin an attractive and tractable biomarker for large epidemiologic studies, such as its long half-life, high *ex vivo* stability, and minimal diurnal variability [8],[9].

While adiponectin levels are highly heritable (30–70%) [10],[11],[12], several well-designed studies have shown variable association between common polymorphisms in the adiponectin gene (*ADIPOQ*), possibly due to small sample sizes and different panels of single nucleotide polymorphisms (SNPs), ethnicities and clinical outcomes [12],[13],[14]. This has lead some observers to call for a more complete and systematic characterization of the genetic determinants of adiponectin levels [12].

Our study therefore sought to address 2 questions: first, what are the common genetic determinants of adiponectin levels both at *ADIPOQ* and elsewhere? And second, do the variants robustly associated with adiponectin levels influence metabolic traits and risk of metabolic disease?

**Figure 1:**
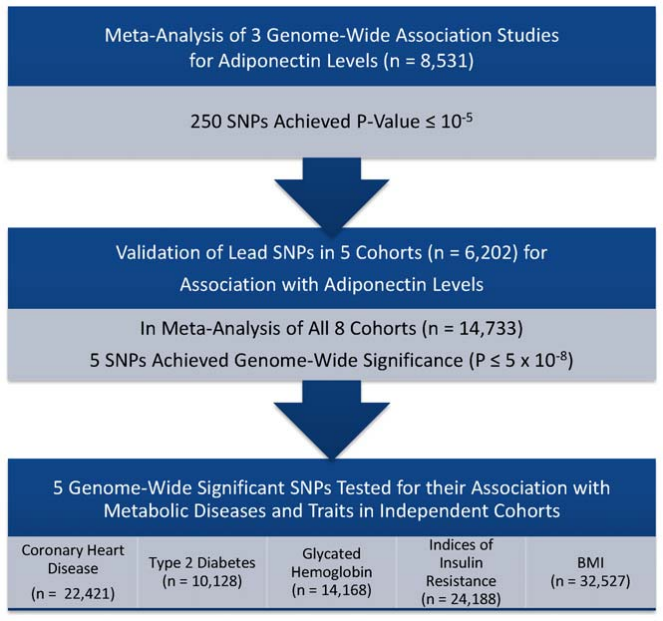
Overall study design.

To comprehensively assess the influence of common genetic variation on circulating adiponectin levels, we undertook a large-scale meta-analysis of 3 genome-wide association studies (GWAS) for circulating adiponectin levels from population-based cohorts (n = 8,531 participants). From this first stage, we chose SNPs most strongly associated with adiponectin levels (P<10^−4^, n = 250), and tested these for their association with adiponectin in 5 additional population-based cohorts (n = 6,202). The 5 SNPs which achieved genome-wide significance in the combined stage were then tested for their association with: type 2 diabetes mellitus (T2D) in the Diabetes Genetics Replication And Meta-analysis (DIAGRAM) consortium [15] (n = 10,128); indices of insulin resistance in the Meta-Analysis of Glucose and Insulin-related traits Consortium (MAGIC) [16] (n = 24,188); risk of coronary heart disease (CHD) in a consortium of 8 cohorts with available genome-wide association data (n = 22,421); and body mass index (BMI) in the Genetic Investigation of Anthropometric Traits (GIANT) consortium (Text S1) [17],[18] (n = 32,527) (Figure 1).

## Results

### Genome-Wide Association Study for Circulating Adiponectin Levels

To identify genetic variants influencing adiponectin levels, we performed a GWAS utilizing information from population-based cohorts including, in total, 14,733 subjects of European descent (Table 1). We identified 5 variants at 2 loci that achieved genome-wide significance (P≤5×10^−8^) for their relationship with circulating adiponectin levels (Table 2). The SNP most strongly associated with circulating adiponectin levels lies 30 kb upstream of the *ADIPOQ* locus (rs266717; P-combined = 9.2×10^−19^) (Table 2, Figure S1, Figure S2). In total, 4 SNPs at the *ADIPOQ* locus demonstrated genome-wide significant associations with circulating adiponectin. All 8 studies contributed to these genome-wide significant associations, with the exception of rs6444175, which demonstrated some heterogeneity across cohorts (Table 2).

**Table 1:** Participant characteristics (n total for all cohorts = 14,733).

	Study	Number of Subjects (% Women)	Method of Adiponectin Measurement	Adiponectin (µg/ml) (SD)	Adiponectin Males (µg/ml) (SD)	Adiponectin Females (µg/ml) (SD)	Age (SD)	BMI (SD)
**Discovery Samples**	**TwinsUK**	1399 (100)	ELISA†	8.1 (3.9)	-	8.1 (3.9)	48.5 (13.1)	25.1 (4.7)
	**GEMS**	1751 (59.3)	ELISA†	6.8 (4.8)	5.8 (4.0)	8.3 (5.5)	52.5 (9.5)	28.5 (3.6)
	**CoLaus**	5381 (47.8)	ELISA†	10.1 (8.1)	7.4 (5.4)	12.4 (9.4)	53.2 (10.8)	25.8 (4.6)
**Replication Samples**	**BLSA**	562 (52.9)	RIA†	13.4 (8.5)	11.5 (7.7)	15.4 (8.9)	67.9 (13.8)	26.8 (4.5)
	**EPIC-Norfolk**	970 (35.5)	ELISA*	6.9 (3.9)	5.6 (2.7)	9.1 (4.6)	62.1 (8.2)	28.3 (4.2)
	**Framingham**	2228 (54.6)	ELISA*	10.5 (6.4)	7.6 (4.5)	13.0 (6.8)	60.4 (9.5)	27.8 (5.0)
	**InCHIANTI**	1027 (54.7)	RIA†	13.5 (9.8)	10.5 (7.6)	15.9 (10.8)	67.6 (15.3)	27.1 (4.1)
	**ALSPAC**	1415 (51.3)	ELISA*	13.1 (5.3)	12.8 (5.1)	13.3 (5.5)	9.9 (0.3)	17.7 (2.9)

*: Plasma.

**†:** Serum.

SD: Standard Deviation, GEMS: Genetic Etiology of Metabolic Syndrome, BLSA: Baltimore Longitudinal Study of Aging, EPIC-Norfolk: European Prospective Investigation of Cancer-Norfolk, ALSPAC: Avon Longitudinal Study of Parents and Children, ELISA: Enzyme-Linked Immunosorbent Assay, RIA: Radio-Immunoassay, BMI: Body Mass Index.

**Table 2:** Relationship of SNPs achieving genome-wide significance for their association with adiponectin levels (n = 14,733 from the 8 studies in Table 1).

Locus	Chr	SNP	Allele (A1*/A2)	MAF	Effect Size on Ln- Transformed Adiponectin (95% CI)	Change in Adiponectin (µg/ml) for Each Effect Allele	P-Value	Q-Test P-Value
*ARL15*	5	rs4311394	G*/A	0.41	−0.04 (−0.06, −0.03)	0.96	2.9E-08	0.38
*ADIPOQ*	3	rs6444175	G/A*	0.28	−0.08 (−0.1, −0.07)	0.92	1.2E-21	0.005
*ADIPOQ*	3	rs266717	T/C*	0.48	0.07 (0.05, 0.09)	1.07	9.2E-19	0.67
*ADIPOQ*	3	rs1426810	G*/A	0.42	0.07 (0.05, 0.08)	1.07	2.2E-18	0.15
*ADIPOQ*	3	rs1648707	C*/A	0.07	−0.06 (−0.07, −0.04)	0.94	3.0E-12	0.42

A1 = Effect Allele.

*: Minor Allele. Effect Size is the change in Natural Log-Transformed adiponectin levels per effect allele.

Chr: Chromosome, SNP: Single Nucleotide Polymorphism, MAF: Minor Allele Frequency, CI: Confidence Interval.

**Figure 2:**
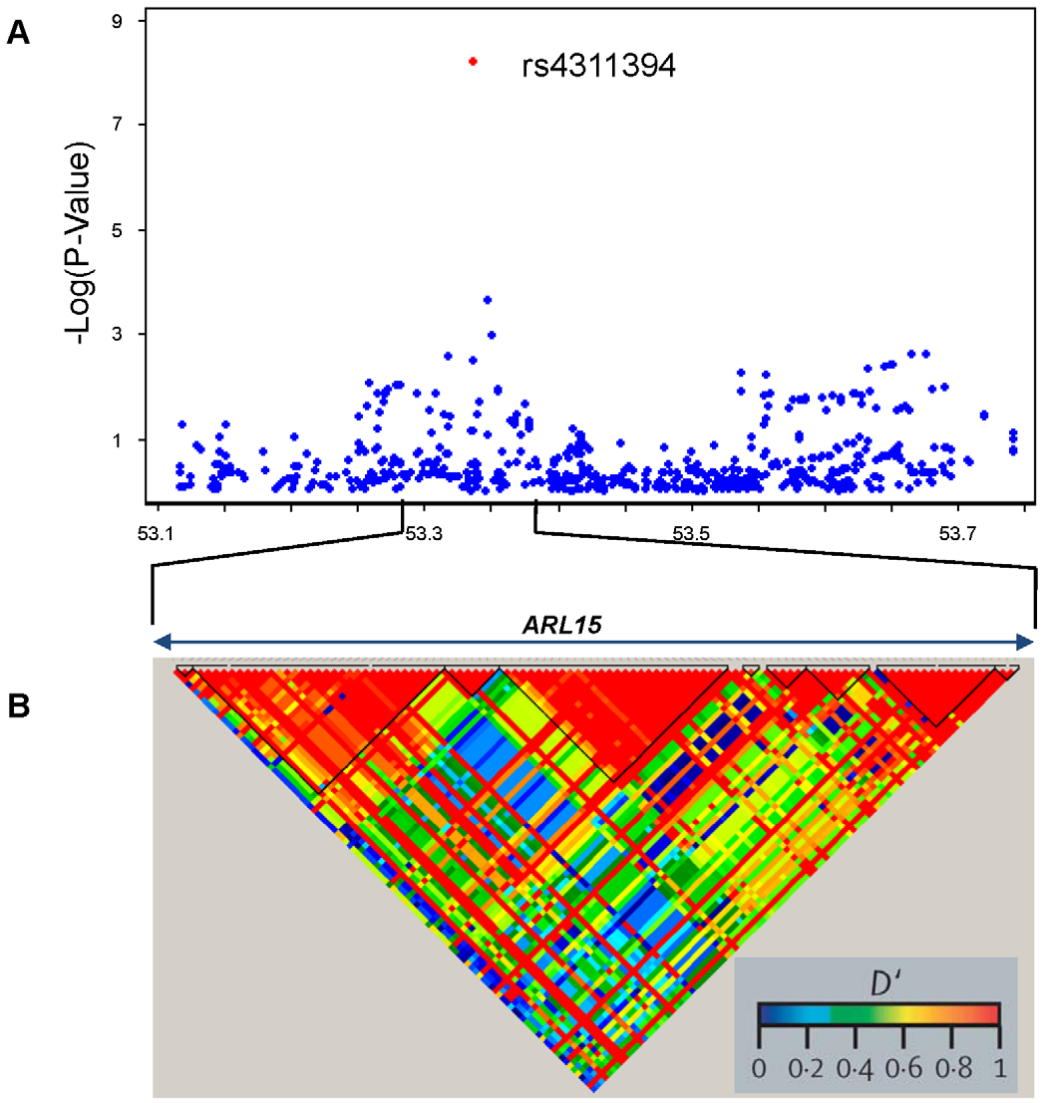
Association between SNPs near *ARL15* and adiponectin levels. (A) −Log(P-value) measures for association between single nucleotide polymorphisms (SNPs) and chromosomal position. (B) Linkage disequilibrium in GOLD heat map Haploview 4.0 color scheme, CEPH (Centre d'Étude du Polymorphisme Humain) population. The x axis represents genomic position in Mb (A) and in kb (B). All P-values are derived from the discovery meta-analysis of CoLaus, TwinsUK, and Genetic Etiology of Metabolic Syndrome (GEMS) cohorts, except that for the lead SNP, rs4311394 (in red), which is derived from the combined P-value from the CoLaus, TwinsUK, GEMS, Framingham, InCHIANTI, Baltimore Longitudinal Study of Aging (BLSA), Avon Longitudinal Study of Parents and Children (ALSPAC), and European Prospective Investigation of Cancer-Norfolk (EPIC-Norfolk) cohorts.

Our results also identified a novel intronic SNP (rs4311394) located in the *ARL15* (ADP-ribosylation factor-like 15) gene whose G allele was robustly associated with decreased adiponectin levels (P = 2.9×10^−8^) (Table 2, Table S3, Figure 2). ARL15 is an ADP-ribosylation factor-like GTP-binding protein, whose function is unknown, yet belongs to a family of proteins involved in intracellular vesicle trafficking [19].

### Association with Metabolic Disease and Metabolic Traits

Since glycemia, T2D and CHD have been correlated with adiponectin levels, we tested whether genome-wide significant SNPs for adiponectin levels were associated with glycemia, indices of insulin resistance, and risk of T2D and CHD. Since 5 SNPs (which, due to linkage disequilibrium, represented 4.59 independent statistical tests [see Methods]) were tested for their association with T2D, CHD and metabolic traits, we employed a conservative Bonferroni-corrected threshold of α = 0.011 (where 0.011 = 0.05/4.59) to declare statistical significance for these metabolic diseases and traits. None of the SNPs at the *ADIPOQ* locus demonstrated a robust relationship with T2D, CHD, homeostasis model assessment insulin resistance (HOMA-IR), homeostasis model assessment beta-cell function (HOMA-B) or BMI (Table 3, Table 4, Table S4). However rs1648707, at *ADIPOQ*, was associated with a non-statistically significant trend for its relationship with CHD (P = 0.04) and T2D (P = 0.046).

**Table 3:** Association of genome-wide significant SNPs with risk of type 2 diabetes mellitus (T2D) and coronary heart disease (CHD) (n = 10,128 for T2D; n = 22,421 for CHD).

Locus	SNP	Effect Allele	Odds Ratio (95% CI) for T2D	P-Value for T2D	Odds Ratio (95% CI) for CHD	P-Value for CHD
***ARL15***	rs4311394	G	1.11 (1.03, 1.18)	0.0032	1.12 (1.06, 1.17)	8.5×10^−6^
***ADIPOQ***	rs6444175	G	0.99 (0.93, 1.07)	0.86	0.97 (0.93, 1.01)	0.14
***ADIPOQ***	rs266717	T	1 (0.94, 1.07)	0.98	0.98 (0.94, 1.02)	0.29
***ADIPOQ***	rs1426810	G	1.01 (0.94, 1.07)	0.86	0.96 (0.92, 0.998)	0.04
***ADIPOQ***	rs1648707	C	1.06 (1, 1.13)	0.046	1.05 (1.003, 1.09)	0.04

SNP: Single Nucleotide Polymorphism, CI: Confidence Interval.

**Table 4:** Association of genome-wide significant SNPs with indices of insulin homeostasis.

Locus	SNP	Effect Allele	Insulin Effect Size (95% CI) [n = 24,616]	Insulin P-Value	HOMA-IR Effect (95% CI) [n = 24,188]	HOMA-IR P-Value	HOMA-B Effect (95% CI) [n = 24,130]	HOMA-B P-Value
***ARL15***	rs4311394	G	0.014 (0.005, 0.023)	2.0×10^−3^	0.012 (0.002, 0.021)	0.01	0.009 (0.001, 0.017)	0.02
***ADIPOQ***	rs6444175	G	0.002 (−0.006, 0.011)	0.62	0.005 (−0.004, 0.014)	0.30	0.003 (−0.005, 0.01)	0.45
***ADIPOQ***	rs266717	T	−0.003 (−0.011, 0.005)	0.46	−0.001 (−0.01, 0.007)	0.76	−0.006 (−0.013, 0.001)	0.08
***ADIPOQ***	rs1426810	G	0 (−0.008, 0.008)	0.99	0.001 (−0.007, 0.01)	0.81	−0.002 (−0.009, 0.005)	0.63
***ADIPOQ***	rs1648707	C	0.009 (0.001, 0.017)	0.032	0.007 (−0.001, 0.016)	0.10	0.003 (−0.004, 0.01)	0.41

SNP: Single Nucleotide Polymorphism, CI: Confidence Interval, HOMA-IR: Homeostasis Model Assessment Insulin Resistance, HOMA-B: Homeostasis Model Assessment Beta-Cell Function.

In contrast, the risk allele rs4311394-G at *ARL15*, which was associated with lower adiponectin levels, was also associated with: an increased risk of CHD in a consortium of 7 CHD cohorts (Odds ratio [OR] = 1.12, [95% Confidence Interval [CI]: 1.06, 1.17], P = 8.5×10^−6^, n = 22,421); an increased risk of T2D in the DIAGRAM consortium [15] (OR = 1.11 [95% CI: 1.03, 1.18], P = 3.2×10^−3^, n = 10,128); and higher glycated hemoglobin in the European Prospective Investigation of Cancer-Norfolk (EPIC-Norfolk) cohort (0.025% per G allele [95% CI: 0.01, 0.04], P = 5.0×10^−4^, n = 14,168) (Table 3). In the MAGIC consortium [16], the rs4311394-G allele was associated with increased levels of fasting insulin (P = 2.3×10^−3^, n = 24,614), and demonstrated non-significant trends towards higher HOMA-IR (P = 0.01, n = 24,188) and HOMA-B (P = 0.02, n = 24,130) (Table 4). In the GIANT consortium [17], the same allele demonstrated a modest and non-significant association with decreased BMI (P = 0.016, n = 32,527) (Table S4), indicating that the disease and metabolic trait associations of rs4311394-G are unlikely to be mediated through an increase in BMI.

Thus, in sum, the G allele at rs4311394 was consistently associated with an increased risk of T2D and CHD, as well as deleterious changes in the 5 metabolic traits tested.

### Expression Studies

**Figure 3:**
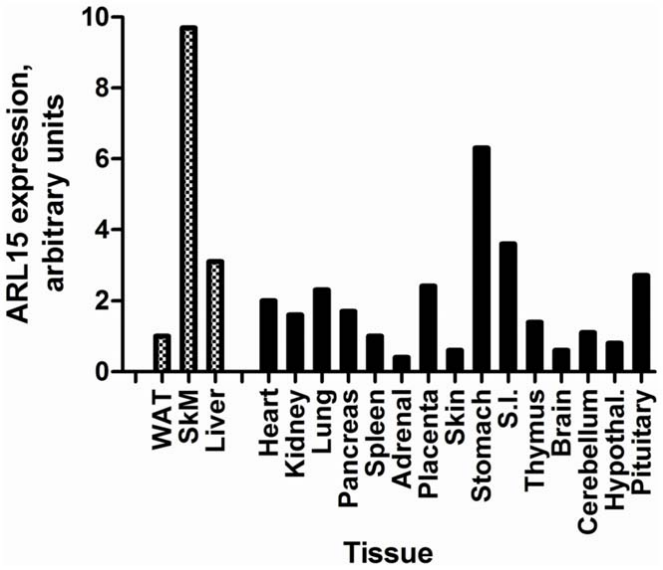
Tissue distribution of ARL15 expression. mRNA levels determined by quantitative real-time PCR in a panel of human tissues.

**Figure 4:**
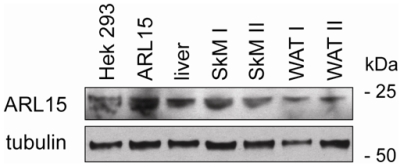
Western blot showing ARL15 expression in insulin-responsive tissues in humans with α-tubulin as a loading control. HEK293 = untransfected HEK293 cells; ARL15 = HEK293 cells transiently expressing wild type human ARL15. SkM = skeletal muscle; WAT = white adipose tissue.

Since the function and distribution of *ARL15* expression is unknown, we assessed the level of *ARL15* mRNA expression in human tissues using quantitative real-time PCR across a wide set of human tissues. We identified that *ARL15* was expressed most abundantly in skeletal muscle at a level 4-fold that of the mean of all other tissues, with adipose expression detectable but low (Figure 3). Using biopsied tissue from insulin-sensitive tissues (liver, skeletal muscle and adipose tissue) in healthy volunteers, immunoblots confirmed ARL15 expression in skeletal muscle, although it was detectable in all 3 tissues (Figure 4).

## Discussion

By conducting a GWAS for the adipocyte-derived protein adiponectin, we have identified a novel susceptibility variant in *ARL15*, which is associated with lower adiponectin levels and increased risk of T2D and CHD. Our results also help clarify which variants at *ADIPOQ* influence adiponectin levels, thus expanding our understanding of the adiponectin pathway.

*ARL15* is widely expressed [20]. However its function is unknown, and there have been no phenotypes previously associated with this gene. Based on its predicted protein sequence, ARL15 is structurally similar to ADP-ribosylation factors and Ras-related GTP-binding proteins which play key roles in the regulation of intracellular vesicle trafficking [19], and which have been specifically implicated in insulin signaling and insulin-stimulated glucose transport [21],[22],[23],[24]. Our preliminary data demonstrate that *ARL15* is expressed in insulin-responsive tissues, including adipose tissue. Interestingly, expression was highest in skeletal muscle, which is the main site of insulin-mediated glucose disposal, but which does not synthesize adiponectin. Thus, *ARL15* is a good candidate to be involved in cellular insulin resistance and/or adiponectin trafficking and secretion. Its implication in metabolic diseases by a non-hypothesis-based genetic approach provides strong impetus for further functional studies.

Our study sheds further light on the role of *ADIPOQ* SNPs on adiponectin levels — which has been the source of several inconsistent reports [12],[13],[14],[25] — since we have systematically tested all common HapMap CEPH (Centre d'Étude du Polymorphisme Humain) SNPs through genotyping and imputation across the *ADIPOQ* locus in 14,733 individuals (Figure S2). Among the SNPs previously associated with adiponectin levels at *ADIPOQ*, the rs1648707 SNP achieved genome-wide significance in our analysis for adiponectin. rs1648707 is in moderate linkage disequilibrium with rs266729 (r^2^ = 0.74), which has previously been associated with adiponectin levels, but not consistently with T2D [12]. We did not assess rare variants, and were thus unable to test the association of rs17366743 (minor allele frequency = 0.075) with adiponectin levels, which has been previously associated with T2D and with fasting glucose, but not with adiponectin levels [13].

Interestingly, *ADIPOQ* SNPs that showed genome-wide significant associations with adiponectin levels did not show associations with T2D or CHD. This raises the question of how ARL15 interacts with adiponectin to influence disease risk. The demonstrated relationship of *ARL15* with the metabolic traits and diseases may represent adiponectin-independent effects of *ARL15* — a hypothesis that could be tested by adjusting the relationship between *ARL15* and CHD or T2D for adiponectin levels (which was not possible in this study, as the disease cohorts had no measured adiponectin levels). Alternatively, recent evidence suggests that adiponectin may be influenced directly by insulin exposure [26]–[35], allowing adiponectin to act as a surrogate marker for integrated total insulin exposure as a result of its stable half-life and relatively low diurnal variability. Consequently, *ARL15* may be an upstream mediator of the relationship between insulin and adiponectin, and may thus impact upon T2D and CHD through an insulin-dependent pathway which involves, but is not entirely dependent upon, adiponectin. In addition, since we demonstrated that the *ARL15* variant was associated with adiponectin levels across all age ranges, including children in the Avon Longitudinal Study of Parents and Children (ALSPAC) cohort, this variant likely affects lifelong adiponectin levels, which may influence its relationship with T2D and CHD.

In conclusion, this study expands our understanding of the genetic influences on adiponectin levels. We have implicated a novel locus, *ARL15*, in the regulation of adiponectin levels and clarified the role of variants near *ADIPOQ* on adiponectin levels. Finally, we provide further evidence that the variant at *ARL15* may influence risk of T2D and CHD, thus providing impetus for further study of *ARL15*.

## Methods

We undertook a GWAS to detect SNPs which were associated with adiponectin, and tested the physiologic and clinical relevance of these SNPs by assessing their association with indices of glucose homeostasis and BMI in European populations, and with T2D and CHD in large clinical cohorts (Figure 1).

### Ethical Considerations

All studies including biopsy of liver, skeletal muscle or adipose tissue from healthy volunteers for immunoblotting studies were approved by institutional ethics review committees at the relevant organizations. All participants provided informed written consent.

### Study Populations

The first stage of the GWAS for adiponectin levels was performed in 3 population-based cohorts utilizing subjects of self-described European ancestry, which were not selected for diabetes, heart disease or any metabolic trait (Table 1). The discovery cohorts included CoLaus [36], TwinsUK [37],[38], and Genetic Etiology of Metabolic Syndrome (GEMS) [39]. Participants of the CoLaus study were individuals of European ancestry, randomly selected from 56,694 permanent residents of Lausanne, Switzerland, between the ages of 35 and 75 years. Recruitment took place between April 2003 and March 2006. TwinsUK is a population-based sample of British twins, which is representative of the general United Kingdom population, and is extensively phenotyped for aging-related traits [40]. GEMS is a case-control study of dyslipidemic individuals between the ages of 20 and 65 years. Cases and controls were matched based on gender and recruitment site. The GEMS and CoLaus studies were sponsored in part by GlaxoSmithKline. All participants were informed of this sponsorship, and consented for the use of their data and biologic samples by GlaxoSmithKline and its subsidiaries.

The validation cohorts included the Framingham Offspring Study (FOS) [13], Baltimore Longitudinal Study of Aging (BLSA) [41], InCHIANTI [42],[43], ALSPAC [44] and EPIC-Norfolk [45]. The FOS is a population-based sample of residents of Framingham, Massachusetts. Adiponectin was measured at exam 7 (1998–2002). BLSA is an observational study that began in 1958 to study normative aging in a cohort of healthy persons 17 years of age and older at study entry. InCHIANTI is a population-based cohort designed to study aging-related traits and disease from the Chianti geographic region (Tuscany, Italy). ALSPAC is a population-based birth cohort study consisting initially of over 13,000 women and their children recruited in the county of Avon, UK, in the early 1990s. The EPIC-Norfolk cohort is a British population-based study of white persons recruited from Norfolk, UK, between 1993 and 1997. All individuals in all replication cohorts were of self-described European descent.

### Phenotyping and Genotyping for Metabolic Traits, T2D, and CHD

Only the SNPs which achieved genome-wide significance for adiponectin levels in the combined analysis of data from all 8 cohorts were assessed for their relationship with adiposity-driven diseases and traits, which included: T2D, CHD, fasting glucose, glycated hemoglobin, BMI and insulin, as well as measures of insulin resistance (HOMA-IR) and beta-cell function (HOMA-B) estimated by the homeostasis model [46].

T2D risk was estimated from the DIAGRAM consortium (a meta-analysis of 3 T2D genome-wide association scans [http://www.well.ox.ac.uk/DIAGRAM/], which included 4,107 T2D cases and 5,187 controls). The 3 populations were the Wellcome Trust Case Control Consortium (WTCCC), the Finland-United States Investigation of NIDDM [Non-Insulin-Dependent Diabetes Mellitus] Genetics (FUSION), and the Diabetes Genetics Initiative (DGI). A full description of this meta-analysis is available elsewhere [15],[47].

The association between susceptibility alleles and fasting glucose, insulin and measures of insulin resistance and beta-cell function were tested in MAGIC [16]. This consortium includes data from 36,610 individuals of European descent who were included in 4 distinct consortia: [a] The European Network for Genetic and Genomic Epidemiology (ENGAGE) project, combining data from deCODE, Northern Finland Birth Cohort 1966, Netherlands Twins Register/Netherlands Study of Depression and Anxiety and the Rotterdam study; [b] the GEMS study, which includes data from the CoLaus and TwinsUK scans; [c] DFS, which includes the DGI, FUSION and SardiNIA scans; and [d] the Framingham Heart Study. Details of all of these studies, phenotyping and genotyping protocols have been published previously [16].

The association between susceptibility alleles and CHD was tested in 8 cohorts (n = 22,421). These cohorts included PennCath [48], MedStar, the Ottawa Heart Study [49], the WTCCC coronary heart disease (CAD) study [50],[51], a case-control study of CHD nested in the EPIC-Norfolk cohort comprising participants with available genome-wide data [52], German Myocardial Infarction Family Study (GerMIFS) I and GerMIFS II [50],[53], and the Rotterdam Study [54] (Table S2). The rs4311394 SNP was assessed by imputation in the GerMIFS I cohort, and did not meet quality control criteria. Thus, results for this SNP are reported for all cohorts except GerMIFS I (Figure S3). All other SNPs were assessed in all cohorts.

Associations with BMI were tested in the GIANT consortium [17],[18], which encompasses 15 cohorts of 32,527 individuals of European descent. It has been described in detail previously, including information on genotyping and phenotyping [17].

### Genotyping

Table S1 outlines the genotyping methods used for each cohort, individual and SNP exclusion thresholds, and imputation algorithms. For the CoLaus and GEMS studies, genotypes were obtained using the Affymetrix Genechip Human Mapping 500k array with the Bayesian Robust Linear Modeling using Mahalanobis distance (BRLMM) algorithm [52]. The TwinsUK samples were genotyped using the Illumina calling algorithm on the Illumina HumanHap300, HumanCNV370 Duo and HumanHap 550 [40]. The FOS employed the Affymetrix 500k and MIPS 50k genotyping arrays. Both the BLSA and InCHIANTI cohorts used the Illumina Human Hap 550 genotyping arrays, while the Illumina Human Hap300 array was used in the ALSPAC cohort. Targeted genotyping was performed in the EPIC-Norfolk cohort using TaqMan SNP genotyping assay (Applied Biosystems, Warrington, UK) according to the manufacturer's protocol. Genotype frequencies were in Hardy Weinberg Equilibrium (HWE) (P>0.50), call rates were >94% and concordances were >98% for the TaqMan assay.

### Adiponectin Measurement

The TwinsUK and EPIC-Norfolk cohorts measured adiponectin levels with an in-house 2-site enzyme-linked immunosorbent assay (ELISA) using antibodies and standards from R&D Systems Europe (Abingdon, Oxford, UK) in plasma. The day-to-day coefficients of variation (CV) for adiponectin were 5.4%, 5.2%, and 5.8% at a concentration of 3.6 µg/ml, 9.2 µg/ml, and 15.5 µg/ml, respectively [38]. The FOS, CoLaus and GEMS measured adiponectin using the ELISA assay (R&D Systems, Minneapolis, Minnesota, United States of America; Intra-assay CV: 5.8%) [13]. Importantly, while CoLaus and GEMs measured adiponectin in plasma, the FOS measured adiponectin in serum. The ALSPAC cohort measured adiponectin using a commercially available ELISA kit (R&D systems, Oxon, UK) previously validated against the corresponding radio-immunoassay (RIA). The inter-assay CV for this adiponectin assay was <7.5%. The InCHIANTI and BLSA studies measured adiponectin levels using the adiponectin RIA assay of Linco Research (St. Charles, Missouri, USA). The detectable ranges for the RIA assay used in InCHIANTI and BLSA are 0.78 µg/ml–200 µg/ml.

### Expression Experiments

Relative levels of *ARL15* mRNA in human tissues were assessed by quantitative real-time PCR of a commercially available human tissue panel of RNA (AMS Biotechnology, Abingdon, UK). 500 ng of RNA were reverse-transcribed using 125 ng of random hexamers and 500 µM deoxynucleotide triphosphates (dNTPs) (both from Promega, Madison, Wisconsin, USA) and 500 ng of Superscript III reverse transcriptase (Invitrogen). Gene expression was quantified on an ABI7900 Real-Time PCR system (Applied Biosystems, Foster City, California, USA) in TaqMan Mastermix (Applied Biosystems). Primers and probe for *ARL15* were supplied by Applied Biosystems (ABI Hs00219491_m1), and *ARL15* expression was normalized to expression of *PPIA* (Cyclophilin A). *PPIA* primers (5′-ACGGCGAGCCCTTGG-3′ (sense), 5′- TTTCTGCTGTCTTTGGGACCT-3′ (antisense)) and probe (5′-[FAM] CGCGTCTCCTTTGAGCTGTTTGCA[TAMRA]-3′) were synthesized by Sigma-Aldrich. Skeletal muscle biopsies were a gift from Dr Anna Krook, from the Karolinska Institute. Frozen skeletal muscle, liver and white adipose tissue samples were homogenized in lysis buffer (50 mM Tris-HCl, pH8.0, 150 mM NaCl, 1 mM EDTA, 1% (v/v) Triton X-100, and Complete Protease Inhibitor Cocktail [Roche]), and cell debris removed by centrifugation. Cleared supernatants were boiled in sodium dodecyl sulphate (SDS) sample buffer and run on an SDS polyacrylamide gel before transfer to a polyvinylidene difluoride (PVDF) membrane (Amersham) and subsequent immunoblotting with either purified rabbit anti-human ARL15 antibody (Proteintech Group) or anti-α-tubulin antibody (sc-8035; Santa Cruz Biotechnology). Full-length human wild type ARL15 cDNA was purchased from Open Biosystems and subcloned into pCDNA 3.1 (Invitrogen) using the XhoI and HindIII restriction sites. HEK293 cells (American Type Culture Collection [ATCC]) were transiently transfected using the CalPhos Mammalian Tranfection Kit (Clontech) according to the manufacturer's instructions.

### Statistical Methods

In all cohorts, the adiponectin concentrations were natural logarithm transformed to create a normally distributed phenotype. Adiponectin levels were subsequently adjusted for age, sex and BMI — important correlates of adiponectin levels [4],[5]. All results reported for association of genetic variants with adiponectin levels are adjusted for age, sex and BMI. All statistical tests assumed an additive effect of the effect allele. In the TwinsUK cohort, we found that there was little difference when comparing results both adjusted, and unadjusted, for BMI (the Spearman coefficients for the beta coefficients was 0.94 and 1.0 for P-values [P-values for both Spearman coefficients<1×10^−5^]).

The SNPTEST software program [51] was used to perform genome-wide association testing in the GEMS and CoLaus cohorts, while the Merlin software package [55] was used to perform association testing in the TwinsUK cohort. The meta-analysis of the discovery phase cohorts (CoLaus, TwinsUK and GEMS) was performed using Liptak-Stouffer's method for combination of independent tests, where P-values are converted to Z-scores by a standard normal curve and weighted by each study's sample size [56].

All SNPs that achieved a combined P-value of ≤10^−4^ in the meta-analysis (n = 250) were tested for their association in the additional cohorts (InCHIANTI, BLSA, ALSPAC and the Framingham Offspring Cohort). Two SNPs that were not near the *ADIPOQ* locus, and which demonstrated associations of ≤5×10^−7^ with adiponectin levels in the combined analysis, were further verified in an additional replication cohort (EPIC-Norfolk), where association with adiponectin was tested using a generalized linear model. For the quantitative trait analyses, individuals with known T2D were excluded. For the T2D case-control analyses, each SNP was tested for association using a logistic regression analysis, adjusted for age, sex and BMI. All analyses for the EPIC-Norfolk cohort were performed with SAS 9.1 (SAS Institute Inc., Cary, North Carolina, USA). To perform a meta-analysis of all replication and discovery cohorts, we employed inverse-variance techniques in the STATA software package (College Station, Texas, USA).

We declared statistical significance in the GWAS as P≤5×10^−8^, where this threshold is based on a Bonferroni correction of α = 0.05 divided by one million, the estimated number of independent common tests among common SNPs in the CEU population of the HapMap II project [57]. Using this threshold, 5 SNPs achieved genome-wide significance for their relationship with circulating adiponectin levels in the combined analysis of all adiponectin cohorts. These were subsequently tested for their association with glycated hemoglobin, indices of insulin resistance, beta-cell function and risk of T2D and CHD. The number of independent statistical tests represented by these 5 SNPs, accounting for linkage disequilibrium at *ADIPOQ*, was assessed by spectral decomposition of matrices of pairwise linkage disequilibrium between the 4 SNPs at the *ADIPOQ* locus [58]. In total, 3.59 independent statistical tests were performed at this locus, and one at the *ARL15* locus. Thus, statistical significance in the follow-up studies was declared at P≤0.011 (based on a Bonferroni correction of α = 0.05 divided by 4.59, the number of statistically independent SNPs tested in the follow-up analyses).

Since 2 cohorts measured adiponectin concentrations using an RIA method (BLSA and InCHIANTI) whilst all others used an ELISA method, and since one study, ALSPAC, was based on children, rather than adults, we tested for evidence of heterogeneity in the combined analysis using the Q-test P-value [59].

## Supporting Information

Figure S1 Association between SNPs near *ADIPOQ* and Adiponectin levels. (A) −log (P value) measures for association between SNPs and chromosomal position. (B) Entrez Genes. (C) Linkage disequilibrium in GOLD heat map Haploview 4.0 color scheme, CEPH population. The x axis represents genomic position in Mb (A) and in kb (B,C). All P values are derived from the discovery meta-analysis, except for the genome-wide significant SNPs (Table 2), which are derived from the combined P values from all cohorts (displayed in red).
(1.13 MB TIF)

Figure S2 Relationship of genome-wide significant SNPs from the current study with selected previously published SNPs at the *ADIPOQ* locus.
(1.54 MB TIF)

Figure S3 Forest Plot of Association of rs4311394 with Risk of CHD (total n = 22,421).
(0.28 MB TIF)

Table S1. Genotyping information for the adiponectin discovery and replication cohorts.
(0.04 MB DOC)

Table S2 Cohort information, case and control definitions for coronary heart disease cohorts. (A) Cohort information for coronary heart disease cohorts and (B) Case and control definitions for coronary heart disease cohorts.
(0.05 MB DOC)

Table S3 Quality control parameters for rs4311394 at *ARL15* from each cohort involved in the adiponectin GWAS.
(0.03 MB DOC)

Table S4 Relationship of genome-wide significant SNPs with body mass index (BMI) in the GIANT consortium.
(0.03 MB DOC)

Text S1 Genetic Investigation of Anthropometric Traits (*GIANT*) Consortium.
(0.05 MB DOC)
