# Supplementary figures and images for: A Genome-Wide Association Study Reveals Variants in ARL15 that Influence Adiponectin Levels
Source: PLoS Genet. 2009 Dec 11;5(12):e1000768. doi: 10.1371/journal.pgen.1000768 (PMC2781107; doi:10.1371/journal.pgen.1000768)

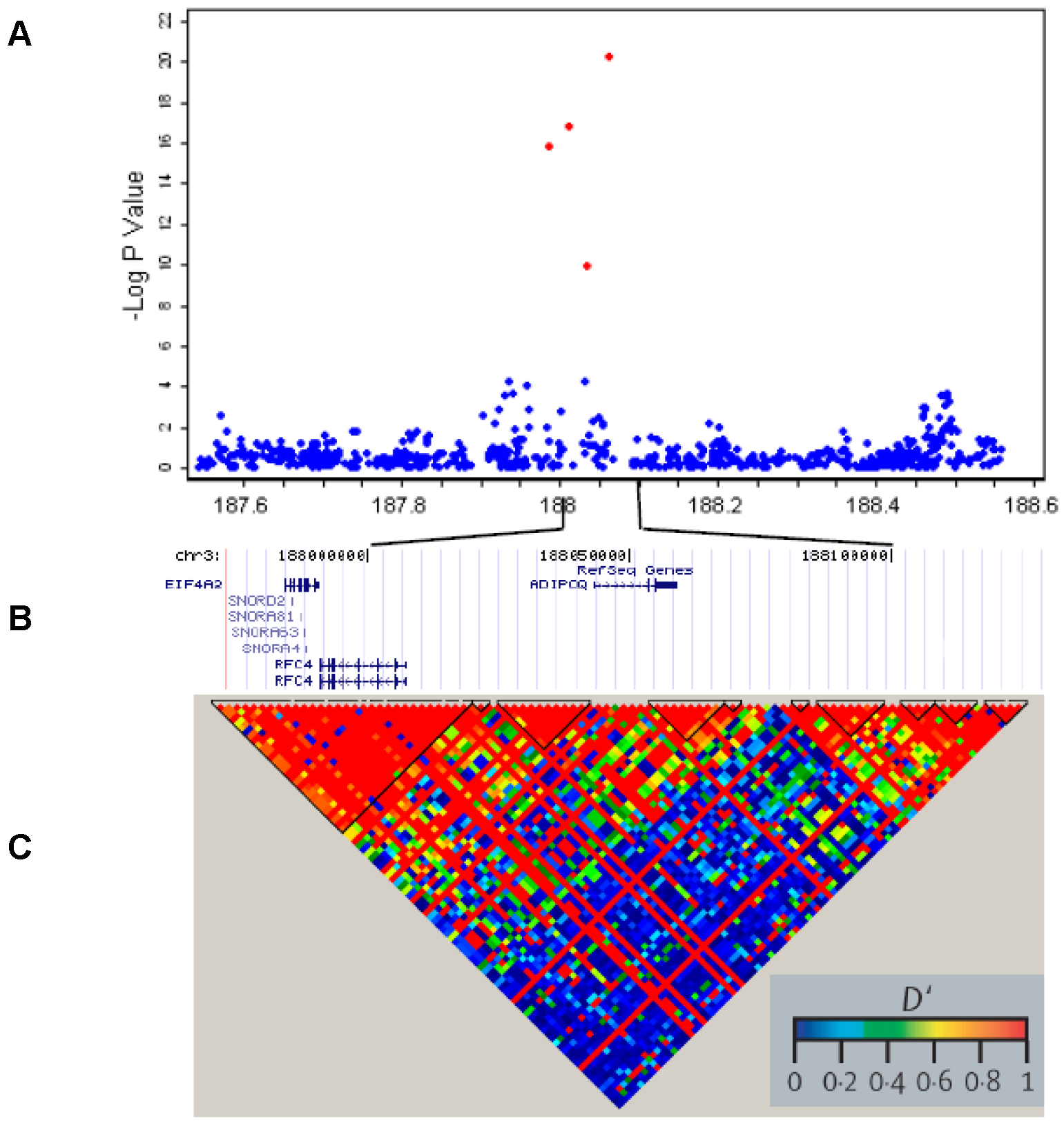

Supplement: Figure S1 — Association between SNPs near ADIPOQ and Adiponectin levels. (A) −log (P value) measures for association between SNPs and chromosomal position. (B) Entrez Genes. (C) Linkage disequilibrium in GOLD heat map Haploview 4.0 color scheme, CEPH population. The x axis represents genomic position in Mb (A) and in kb (B,C). All P values are derived from the discovery meta-analysis, except for the genome-wide significant SNPs (Table 2), which are derived from the combined P values from all cohorts (displayed in red). (1.13 MB TIF) [file pgen.1000768.s001.tif]

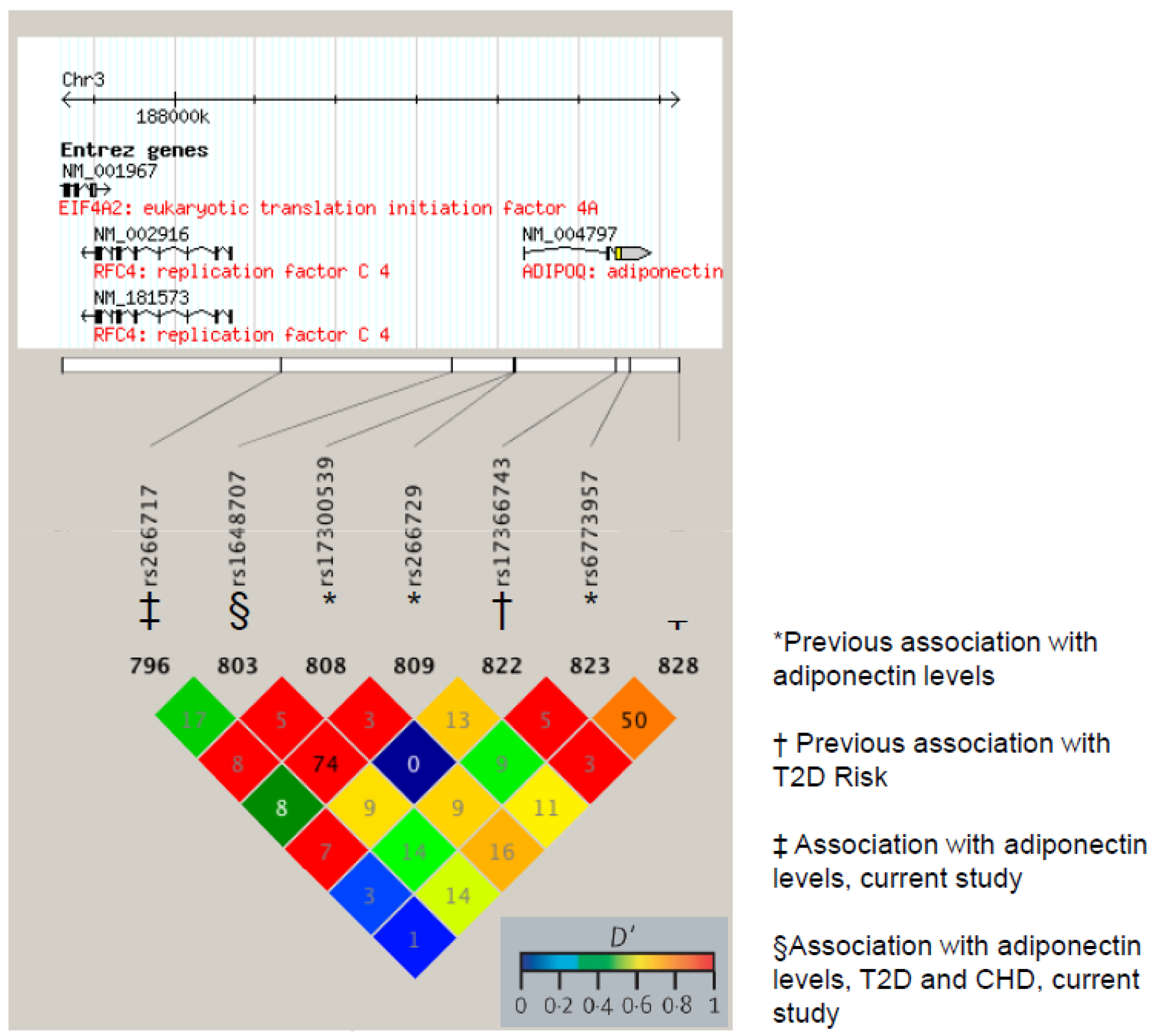

Supplement: Figure S2 — Relationship of genome-wide significant SNPs from the current study with selected previously published SNPs at the ADIPOQ locus. (1.54 MB TIF) [file pgen.1000768.s002.tif]

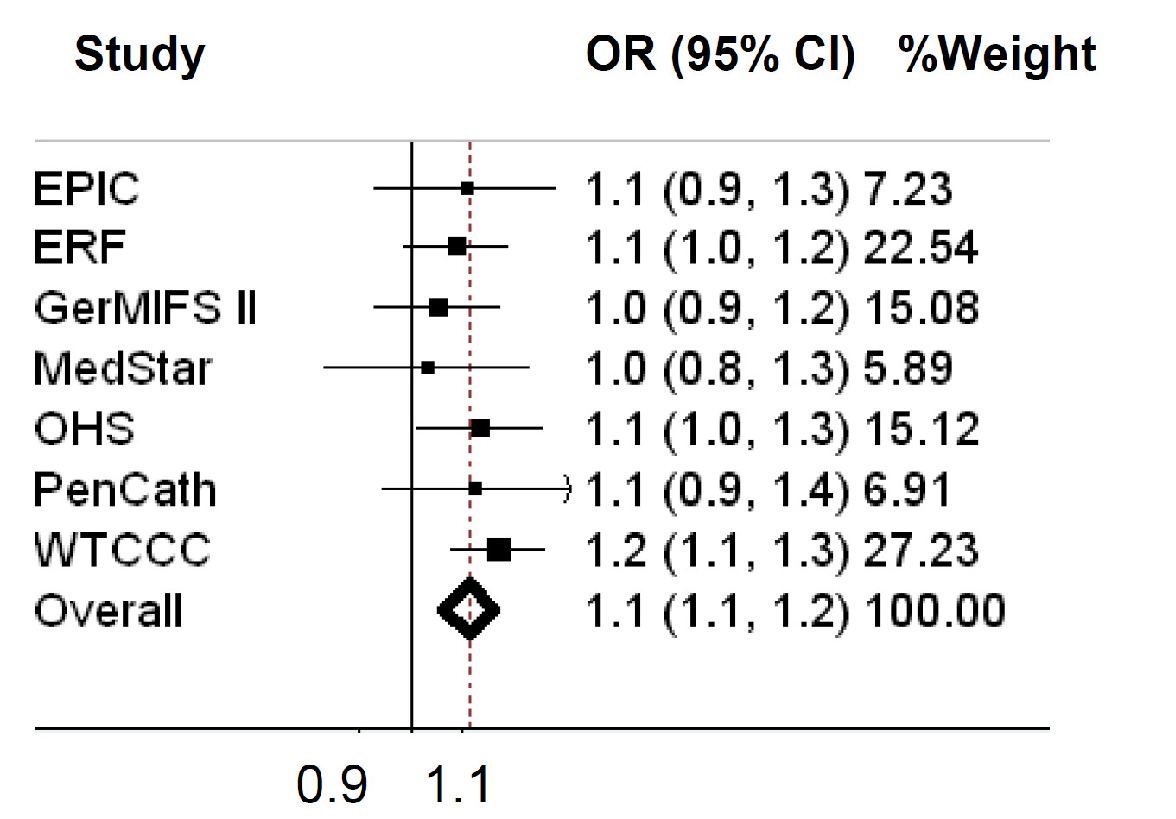

Supplement: Figure S3 — Forest Plot of Association of rs4311394 with Risk of CHD (total n = 22,421). (0.28 MB TIF) [file pgen.1000768.s003.tif]
