# Supplementary material for: A Genome-Wide Association Study Reveals Variants in ARL15 that Influence Adiponectin Levels
Source: PLoS Genet. 2009 Dec 11;5(12):e1000768. doi: 10.1371/journal.pgen.1000768 (PMC2781107; doi:10.1371/journal.pgen.1000768)
Supplement: Table S1. — Genotyping information for the adiponectin discovery and replication cohorts. (0.04 MB DOC) [file pgen.1000768.s004.doc]

|  |  | **Genotyping** | | | **SNPs** | | | **Imputation** | | |  |  |
| --- | --- | --- | --- | --- | --- | --- | --- | --- | --- | --- | --- | --- |
|  | **Study** | **Call Rate** | **Sample QC/ Other Exclusions** | **Platform/ Chip** | **MAF** | **P-Test HWE** | **Included SNPs** | **Method** | **MAF** | **Quality Metric** | **Total # SNPs Analyzed** | **λ_GC_** |
| **Discovery Cohorts** | **TwinsUK** | 95% | 1. autosomal heterozygosity < 0.33 or > 0.37  2. ethnic outliers (using STRUCTURE)  3. missing adiponectin, BMI or age | Illumina HumanHap 300  Illumina HumanCNV370 Duo | ≥ 5% | ≥ 10^-6^ | 307040 | IMPUTE | ≥ 1% | Prop.Inf ≥ 0.4 | 2288471 | 1.001 |
|  | **CoLaus** | 90% | 1. Gender inconsistency with genetic data from x-lined markers  2. Inconsistent genotypes when compared with control markers  3. Duplicates and first and second degree relatives | Affymetrix 500k | ≥ 1% | ≥ 10^-6^ | 390631 | IMPUTE | ≥ 1% | Prop.Inf ≥ 0.4 | 2557249 | 1.01 |
|  | **GEMS** | 90% | 1. Gender inconsistency with genetic data from x-lined markers  2. Inconsistent genotypes when compared with control markers  3. Duplicates and first and second degree relatives | Affymetrix 500k | ≥ 1% | ≥ 10^-6^ | 357170 | IMPUTE | ≥ 1% | Prop.Inf ≥ 0.4 | 2557249 | 1.01 |
| **Replication Cohorts** | **FOS** | 95% | 1. Heterozygosity filter 5 SD from mean (< 25.8% of > 30%)  2. Ethnic outliers (assessed, but none excluded)  3. missing adiponectin, BMI or age  4. > 1000 Mendelian Errors | Affymetrix 500k and MIPS 50k | ≥ 1% | ≥ 10^-6^ | 434050 | MACH | ≥ 1% | MACH R Squared | 250 | 1.027 |
|  | **BLSA** | 99% | 1. Sex misspecification  2. ethnic outliers (using EIGENSTRAT) | Illumina HumanHap 550 | ≥ 1% | ≥ 10^-4^ | 501764 | MACH | > 1% | r^2^ > 0.3 | 250 | 1.034 |
|  | **InCHIANTI** | ≥ 98% | Gender mismatch and missing phenotypes/covariates | Illumina HumanHap 550 | ≥ 1% | 0.0001 | 496032 | IMPUTE | > 1% | Prop.Inf ≥ 0.4 | 250 | 1.027 |
|  | **ALSPAC** | 99% | 1. Heterozygosity filter (< 34.3% of > 36.4%)  2. Ethnic outliers via MDS  3. Missing rate > 5%  4. Sex misspecification | Illumina HumanHap 300k | ≥ 1% | ≥ 10^-7^ | 305718 | MACH | > 1% | MACH  r^2^ > 0.25 | 248 | 1.004 |

λ_GC_: Genomic Inflation Factor, MAF: Minor Allele Frequency, QC: Quality Criteria, HWE: Hardy Weinberg Equilibrium, SNPs: Single Nucleotide Polymorphisms. Note that the EPIC-Norfolk performed targeted genotyping of replication SNPs, as described in the main text, rather than use of genome-wide genotyping. Note that only SNPs achieving P-values of ≤ 10^-4^ (n = 250) were tested for their association with adiponectin in the Replication Cohorts.
