# Supplementary material for: A Genome-Wide Association Study Reveals Variants in ARL15 that Influence Adiponectin Levels
Source: PLoS Genet. 2009 Dec 11;5(12):e1000768. doi: 10.1371/journal.pgen.1000768 (PMC2781107; doi:10.1371/journal.pgen.1000768)
Supplement: Table S2 — Cohort information, case and control definitions for coronary heart disease cohorts. (A) Cohort information for coronary heart disease cohorts and (B) Case and control definitions for coronary heart disease cohorts. (0.05 MB DOC) [file pgen.1000768.s005.doc]

A

|  | OHS | WTCCC | EPIC-Norfolk | GerMIFS I | GerMIFS II | PennCath | MedStar | Rotterdam Study |
| --- | --- | --- | --- | --- | --- | --- | --- | --- |
| Number of Cases/Controls | 1542/1455 | 1926/2938 | 421/2698 | 875/1644 | 1222/1298 | 933/468 | 1064/483 | 928/5046 |
| Mean Age Cases (SD) /Controls (SD) | 48.7 (7.3) / 75.0 (5.0) | Men 49.4 (7.7), Women 51.0 (7.4) / Mean age not available for controls | 64.1 (7.4) / 58.0 (8.8) | 50.2 (7.9) / 62.5 (10.1) | 51.3 (7.6) / 51.2 (11.9) | 56.8 (9.2) / 61.7 (9.6) | 55.0 (7.8) / 60.0 (8.9) | 71.7 (8.9) / 69.0 (9.1) |
| % Male Cases/Controls | 75.8/52.0 | 79.3 / 50.0 | 67.0 / 41.1 | 67.5 / 49.2 | 79.7 / 52.1 | 75.7/48.3 | 71.2/51.4 | 55.7 / 37.9 |
| Mean BMI Cases (SD) /Controls (SD) | 28.6 (4.9) / 26.0 (4.0) | - | 29.3 (4.3) / 28.2 (4.7) | 27.5 (3.7) / 28.1 (4.5) | 27.9 (4.0) / 27.4 (4.6) | 29.8 (5.6) / 29.0 (6.4) | 31.4 (7.0) / 31.4 (7.7) | 26.3 (3.6) / 26.3 (3.7) |
| Genotyping Platform | Affymetrix 6.0 | Affymetrix 500k | Affymetrix 500k | Affymetrix 550k | Affymetrix 6.0 | Affymetrix 6.0 | Affymetrix 6.0 | Illumina 550k |
| Reference for Further Information | [1] | [2,3] | [4] | [2,5] | [5,6] | [7] | [7] | [8] |

OHS: Ottawa Heart Study, WTCCC: Wellcome Trust Case Control Consortium, EPIC-Norfolk: European Prospective Investigation of Cancer-Norfolk, GerMIFS: German Myocardial Infarction Family Study, BMI: Body Mass Index, SD: Standard Deviation. Note that BMI data is not available for the WTCCC cohort.

B

| OHS | Cases: Symptomatic CAD before the age of 55 years in males and 65 in females. CAD, defined as greater than 50% stenosis of a coronary artery, was confirmed by coronary angiography. Cases with a history of diabetes were excluded. Controls: Asymptomatic individuals > 65 years for males > 75 years for females |
| --- | --- |
| WTCCC | Cases: Nationally recruited unrelated individuals of European white ethnicity with premature CAD (a validated history of either MI or coronary revascularization before age 66 years) and one or more first degree relatives with CAD. Controls: Unrelated individuals from British 1958 Birth Cohort and UK Blood Services. |
| EPIC-Norfolk | Cases: Individuals of European white ethnicity with genome-wide data available and who have prevalent or incident CAD as either self-reported at baseline healthcheck or death certificate/hospital discharge notes. For the latter, IHD is defined as ICD9 410-414 or ICD10 I20-I25. Controls: Individuals of European white ethnicity with genome-wide data available without prevalent or incident CAD. All participants with prevalent and incident stroke were excluded. |
| GerMIFS I | Cases: Suffered Myocardial Infarction (age of onset < 65 years) having at least one first-degree relative with premature CAD. Controls: Healthy German married-in spouses from the same recruitment centre, and overlapping with the KORA F3 500K sample collection |
| GerMIFS II | Cases: MI prior to the age of 60 years. Moreover, a positive family history for CAD was documented in 59.4 % of cases. Controls: Derived from the MONICA/KORA Augsburg survey S4 1 (n = 820) and the PopGen blood donor sample 2 (PopGen-BSP) (n = 478).  1,222 |
| PennCath | Cases: One or more coronary vessels with ≥ 50% stenosis equally selected for stable CAD cases without history of MI and CAD cases with a history of MI. Controls were aged over 40 for men and 45 in women who showed no angiographic evidence of CAD. |
| MedStar | Cases: One or more coronary vessels with ≥ 50% stenosis equally selected between stable CAD cases without history of MI and CAD cases with a history of MI. Controls were aged over 45 showed no angiographic evidence of CAD. |
| ERF | Incident CHD defined as MI, revascularization or cardiovascular death. |

References

1. McPherson R, Pertsemlidis A, Kavaslar N, Stewart A, Roberts R, et al. (2007) A common allele on chromosome 9 associated with coronary heart disease. Science 316: 1488-1491.

2. Samani NJ, Erdmann J, Hall AS, Hengstenberg C, Mangino M, et al. (2007) Genomewide association analysis of coronary artery disease. N Engl J Med 357: 443-453.

3. Consortium W.T.C.C. (2007) Genome-wide association study of 14,000 cases of seven common diseases and 3,000 shared controls. Nature 447: 661-678.

4. Sandhu MS, Waterworth DM, Debenham SL, Wheeler E, Papadakis K, et al. (2008) LDL-cholesterol concentrations: a genome-wide association study. Lancet 371: 483-491.

5. Fischer M, Broeckel U, Holmer S, Baessler A, Hengstenberg C, et al. (2005) Distinct heritable patterns of angiographic coronary artery disease in families with myocardial infarction. Circulation 111: 855-862.

6. Erdmann J, Großhennig A, Braund P, König I, Hengstenberg C, et al. (2009) New susceptibility locus for coronary artery disease on chromosome 3q22.3. Nature Genetics, In Press.

7. Myocardial Infarction Genetics Consortium, Kathiresan S, Voight BF, Purcell S, Musunuru K, et al. (2009) Genome-wide association of early-onset myocardial infarction with single nucleotide polymorphisms and copy number variants. Nat Genet 41: 334-341.

8. Hofman A, Breteler MM, van Duijn CM, Krestin GP, Pols HA, et al. (2007) The Rotterdam Study: objectives and design update. Eur J Epidemiol 22: 819-829.
