# Supplementary material for: A Genome-Wide Association Study Reveals Variants in ARL15 that Influence Adiponectin Levels
Source: PLoS Genet. 2009 Dec 11;5(12):e1000768. doi: 10.1371/journal.pgen.1000768 (PMC2781107; doi:10.1371/journal.pgen.1000768)
Supplement: Table S3 — Quality control parameters for rs4311394 at ARL15 from each cohort involved in the adiponectin GWAS. (0.03 MB DOC) [file pgen.1000768.s006.doc]

|  | **Cohort** | **MAF** | **% Missing** | **HWE**  **P-Value** | **R-Squared MACH** | **Info from IMPUTE** |
| --- | --- | --- | --- | --- | --- | --- |
| **Genotyped** | **ALSPAC** | 0.26 | 1.1 x 10^-2^ | 0.25 | - | - |
|  | **BLSA** | 0.21 | 1.8 x 10^-3^ | 0.21 | - | - |
|  | **TwinsUK** | 0.24 | 1.3 x 10^-3^ | 0.86 | - | - |
| **Imputed** | **Framingham** | 0.26 | 0 | - | 0.992 |  |
|  | **InCHIANTI** | 0.28 | 3.9 x 10^-3^ | - | - | 0.99 |
|  | **CoLaus** | 0.27 | 2.0 x 10^-4^ | - | - | 0.99 |
|  | **GEMS** | 0.25 | 2.0 x 10^-4^ | - | - | 0.99 |

ALSPAC: Avon Longitudinal Study of Parents and Children, BLSA: Baltimore Longitudinal Study of Aging, GEMS: Genetic Etiology of Metabolic Syndrome, MAF: Minor Allele Frequency, HWE: Hardy Weinberg Equilibrium.
