# Supplementary material for: A Genome-Wide Association Study Reveals Variants in ARL15 that Influence Adiponectin Levels
Source: PLoS Genet. 2009 Dec 11;5(12):e1000768. doi: 10.1371/journal.pgen.1000768 (PMC2781107; doi:10.1371/journal.pgen.1000768)
Supplement: Table S4 — Relationship of genome-wide significant SNPs with body mass index (BMI) in the GIANT consortium. (0.03 MB DOC) [file pgen.1000768.s007.doc]

| **Locus** | **SNP** | **Alleles** | **N** | **Effect** | **P-Value** |
| --- | --- | --- | --- | --- | --- |
| *ARL15* | rs4311394 | A*/G | 32527 | 0.023 | 0.016 |
| *ADIPOQ* | rs6444175 | A*/G | 32521.02 | 0.002 | 0.809 |
| *ADIPOQ* | rs266717 | T*/C | 32512.07 | 0.003 | 0.741 |
| *ADIPOQ* | rs1426810 | A*/G | 32514.4 | 0.003 | 0.764 |
| *ADIPOQ* | rs1648707 | A*/C | 32521.31 | 0.007 | 0.441 |

***Effect Allele. N = Sample Size.** SNP: Single Nucleotide Polymorphism.
