# Supplementary material for: A Genome-Wide Association Study Reveals Variants in ARL15 that Influence Adiponectin Levels
Source: PLoS Genet. 2009 Dec 11;5(12):e1000768. doi: 10.1371/journal.pgen.1000768 (PMC2781107; doi:10.1371/journal.pgen.1000768)
Supplement: Text S1 — Genetic Investigation of Anthropometric Traits (GIANT) Consortium. (0.05 MB DOC) [file pgen.1000768.s008.doc]

**Genetic Investigation of Anthropometric Traits (GIANT) Consortium**

Cristen J. Willer,^1,77,78^ Elizabeth K. Speliotes,^2,3,77,78^ Ruth J. F. Loos,^4,5,77,78^ Shengxu Li,^4,5,77,78^ Cecilia M. Lindgren,^6,21,78^ Iris M. Heid,^7,78^ Sonja I. Berndt,^8^ Amanda L. Elliott,^9,10^ Anne U. Jackson,^1^ Claudia Lamina,^7^ Guillaume Lettre,^9,11^ Noha Lim,^12^ Helen N. Lyon,^3,11^ Steven A. McCarroll,^9,10^ Konstantinos Papadakis,^13^ Lu Qi,^14,15^ Joshua C. Randall,^6^ Rosa Maria Roccasecca,^16^ Serena Sanna,^17^ Paul Scheet,^18^ Michael N. Weedon,^19^ Eleanor Wheeler,^16^ Jing Hua Zhao,^4,5^ Leonie C. Jacobs,^20^ Inga Prokopenko,^6,21^ Nicole Soranzo,^16,22^ Toshiko Tanaka,^23^ Nicholas J. Timpson,^24^ Peter Almgren,^25^ Amanda Bennett,^26^ Richard N. Bergman,^27^ Sheila A. Bingham,^28,29^ Lori L. Bonnycastle,^30^ Morris Brown,^31^ Noël P. Burtt,^9^ Peter Chines,^30^ Lachlan Coin,^32^ Francis S. Collins,^30^ John M. Connell,^33^ Cyrus Cooper,^34^ George Davey Smith,^24^ Elaine M. Dennison,^34^ Parimal Deodhar,^30^ Paul Elliott,^32^ Michael R. Erdos,^30^ Karol Estrada,^20^ David M. Evans^24^ Lauren Gianniny^9^ Christian Gieger^7^ Christopher J Gillson^4,5^ Candace Guiducci^9^ Rachel Hackett,^9^ David Hadley,^13^ Alistair S. Hall,^35^ Aki S. Havulinna,^36^ Johannes Hebebrand,^37^ Albert Hofman,^38^ Bo Isomaa,^39^ Kevin B. Jacobs,^40^ Toby Johnson,^41–43^ Pekka Jousilahti,^36^ Zorica Jovanovic,^5,44^ KayTee Khaw,^45^ Peter Kraft,^46^ Mikko Kuokkanen,^9,47^ Johanna Kuusisto,^48^ Jaana Laitinen,^49^ Edward G. Lakatta,^50^ Jian’an Luan,^4,5^ Robert N. Luben,^45^ Massimo Mangino,^69^ Wendy L. McArdle,^52^ Thomas Meitinger,^53,54^ Antonella Mulas,^17^ Patricia B. Munroe,^55^ Narisu Narisu,^30^ Andrew R. Ness,^56^ Kate Northstone,^52^ Stephen O’Rahilly,^5,44^ Carolin Purmann,^5,44^ Matthew G. Rees,^30^ Martin Ridderstråle,^57^ Susan M. Ring,^52^ Fernando Rivadeneira,^20,38^ Aimo Ruokonen,^58^ Manjinder S. Sandhu,^4,45^ Jouko Saramies,^59^ Laura J. Scott,^1^ Angelo Scuteri,^60^ Kaisa Silander,^47,71^ Matthew A. Sims,^4,5^ Kijoung Song,^12^ Jonathan Stephens,^61^ Suzanne Stevens,^51^ Heather M. Stringham,^1^ Y. C. Loraine Tung,^5,44^ Timo T. Valle,^62^ Cornelia M. Van Duijn,^38^ Karani S. Vimaleswaran,^4,5^ Peter Vollenweider,^63^ Gerard Waeber,^63^ Chris Wallace,^55^ Richard M. Watanabe,^64^ Dawn M. Waterworth,^12^ Nicholas Watkins,^61^ The Wellcome Trust Case Control Consortium,^76^ Jacqueline C. M. Witteman,^38^ Eleftheria Zeggini,^6^ Guangju Zhai,^22^ M. Carola Zillikens,^20^ David Altshuler,^9,10^ Mark J. Caulfield,^55^ Stephen J. Chanock,^8^ I. Sadaf Farooqi,^5,44^ Luigi Ferrucci,^23^ Jack M. Guralnik,^65^ Andrew T. Hattersley,^66^ Frank B. Hu,^14,15^ Marjo-Riitta Jarvelin,^32^ Markku Laakso,^48^ Vincent Mooser,^12^ Ken K. Ong,^4,5^ Willem H. Ouwehand,^16,61^ Veikko Salomaa,^36^ Nilesh J. Samani,^51^ Timothy D. Spector,^22^ Tiinamaija Tuomi,^67,68^ Jaakko Tuomilehto,^67^ Manuela Uda,^17^ André G. Uitterlinden,^20,38^ Nicholas J. Wareham,^4,5^ Panagiotis Deloukas,^16^ Timothy M. Frayling,^19^ Leif C. Groop,^25^ Richard B. Hayes,^8^ David J. Hunter,^9,14,15,46^ Karen L. Mohlke,^70^ Leena Peltonen,^9,16,71^ David Schlessinger,^72^ David P. Strachan,^13^ H-Erich Wichmann,^7,73^ Mark I. McCarthy,^6,21,74,78,79^ Michael Boehnke,^1,78,79^ Inês Barroso,^16,78,79^ Gonçalo R. Abecasis,^18,78,79^ Joel N. Hirschhorn,^3,11,75,78,79^ for the GIANT Consortium^80^

1. Center for Statistical Genetics, Department of Biostatistics, University of Michigan School of Public Health, Ann Arbor, Michigan, United States of America.

2. Division of Gastroenterology, Massachusetts General Hospital, Boston, Massachusetts, United States of America.

3. Metabolism Initiative and Program in Medical and Population Genetics, Broad Institute of Harvard and Massachusetts Institute of Technology, Boston, Massachusetts, United States of America.

4. Medical Research Council Epidemiology Unit, Addenbrooke’s Hospital, Cambridge, United Kingdom.

5. Institute of Metabolic Science, Addenbrooke’s Hospital, Cambridge, United Kingdom.

6. Wellcome Trust Centre for Human Genetics, University of Oxford, Oxford, United Kingdom.

7. Institute of Epidemiology, Helmholtz Zentrum München, Neuherberg, Germany.

8. Division of Cancer Epidemiology and Genetics, National Cancer Institute, National Institutes of Health, Department of Health and Human Services, Bethesda, Maryland, United States of America.

9. Program in Medical and Population Genetics, Broad Institute of Massachusetts Institute of Technology and Harvard, Cambridge, Massachusetts, United States of America.

10. Center for Human Genetic Research, Massachusetts General Hospital, Boston, Massachusetts, United States of America.

11. Program in Genomics and Divisions of Endocrinology and Genetics, Children’s Hospital, Boston, Massachusetts, United States of America.

12. Medical Genetics/Clinical Pharmacology and Discovery Medicine, GlaxoSmithKline, King of Prussia, Pennsylvania, United States of America.

13. Division of Community Health Sciences, St. George’s, University of London, London, United Kingdom.

14. Department of Nutrition, Harvard School of Public Health, Boston, Massachusetts, United States of America.

15. Channing Laboratory, Department of Medicine, Brigham and Women’s Hospital, Boston, Massachusetts, United States of America.

16. Wellcome Trust Sanger Institute, Hinxton, Cambridge, United Kingdom.

17. Istituto di Neurogenetica e Neurofarmacologia, Consiglio Nazionale delle Ricerche, Cagliari, Italy.

18. Department of Epidemiology, University of Texas, M.D. Anderson Cancer Center, Houston, Texas, United States of America.

19. Genetics of Complex Traits, Peninsula Medical School, Exeter, United Kingdom.

20. Department of Internal Medicine, Erasmus Medical Center, Rotterdam, the Netherlands.

21. Oxford Centre for Diabetes, Endocrinology and Metabolism, University of Oxford, Churchill Hospital, Oxford, United Kingdom.

22. Department of Twin Research and Genetic Epidemiology, King’s College London, London, United Kingdom.

23. Clinical Research Branch, National Institute on Aging, Baltimore, Maryland, United States of America.

24. Medical Research Council Centre for Causal Analyses in Translational Epidemiology, Department of Social Medicine, University of Bristol, Bristol, United Kingdom.

25. Lund University Diabetes Centre, Department of Clinical Sciences, Lund University, Malmö, Sweden.

26. Oxford Centre for Diabetes, Endocrinology and Metabolism, University of Oxford, Oxford, United Kingdom.

27. Physiology and Biophysics, University of Southern California School of Medicine, Los Angeles, California, United States of America.

28. Medical Research Council Dunn Human Nutrition Unit, Wellcome Trust/ Medical Research Council Building, Cambridge, United Kingdom.

29. Medical Research Council Centre for Nutritional Epidemiology in Cancer Prevention and Survival,

Cambridge, United Kingdom.

30. National Human Genome Research Institute, Bethesda, Maryland, United States of America.

31. Clinical Pharmacology Unit, University of Cambridge, Addenbrooke’s Hospital, Cambridge, United Kingdom.

32. Department of Epidemiology and Public Health, Imperial College London, London, United Kingdom.

33. British Heart Foundation Glasgow Cardiovascular Research Centre, Faculty of Medicine, University of Glasgow, Glasgow, United Kingdom.

34. Medical Research Council Epidemiology Resource Centre, University of Southampton, Southampton General Hospital, Southampton, United Kingdom.

35. Yorkshire Heart Centre, Leeds General Infirmary, Leeds, United Kingdom.

36. National Institute for Health and Welfare, Unit of Chronic Disease Epidemiology and Prevention, Helsinki, Finland.

37. Department of Child and Adolescent Psychiatry, University of Duisburg‐Essen, Essen, Germany.

38. Department of Epidemiology, Erasmus Medical Center, Rotterdam, the Netherlands.

39. Folkhalsan Research Center, Malmska Municipal Health Care Center and Hospital, Jakobstad, Finland.

40. Bioinformed Consulting Services, Gaithersburg, Maryland 20877, USA.

41. Department of Medical Genetics, University of Lausanne, CH‐1005 Lausanne, Switzerland.

42. University Institute for Social and Preventative Medicine, Centre Hospitalier Universitaire Vaudois (CHUV), Lausanne, Switzerland.

43. Swiss Institute of Bioinformatics, Lausanne, Switzerland.

44. University of Cambridge Metabolic Research Laboratories, Addenbrooke’s Hospital, Cambridge, United Kingdom.

45. Department of Public Health and Primary Care, Institute of Public Health, University of Cambridge, Cambridge, United Kingdom.

46. Program in Molecular and Genetic Epidemiology, Harvard School of Public Health, Boston, Massachusetts, United States of America.

47. National Institute for Health and Welfare, Unit of Public Health Genomics, Helsinki, Finland.

48. Department of Medicine, University of Kuopio, Kuopio, Finland.

49. Finnish Institute of Occupational Health, Oulu, Finland.

50. Laboratory of Cardiovascular Science, Gerontology Research Center, National Institute on Aging, Baltimore, Maryland, United States of America.

51. Department of Cardiovascular Sciences, University of Leicester, Clinical Sciences, Glenfield General Hospital, Leicester, United Kingdom.

52. Avon Longitudinal Study of Parents and Children (ALSPAC), Department of Social Medicine, University of Bristol, Bristol, United Kingdom.

53. Institute of Human Genetics, Helmholtz Zentrum München, Neuherberg, Germany.

54. Institute of Human Genetics, Technical University Munich, Munich, Germany.

55. Clinical Pharmacology, The William Harvey Research Institute, Barts and The London, Queen Mary’s School of Medicine and Dentistry, London, United Kingdom.

56. Department of Oral & Dental Science, University of Bristol, Bristol, United Kingdom.

57. Department of Clinical Sciences, Lund University, Malmö, Sweden.

58. Department of Clinical Chemistry, University of Oulu, Oulu, Finland.

59. Savitaipale Health Center, Savitaipale, Finland.

60. Unitá Operativa Geriatria, Istituto Nazionale Ricovero e Cura per Anziani (INRCA IRCCS), Rome, Italy.

61. Department of Hematology, University of Cambridge/National Health Service Blood & Transplant, Cambridge, United Kingdom.

62. National Institute for Health and Welfare, Unit of Diabetes Prevention, Helsinki, Finland.

63. Department of Internal Medicine, Centre Hospitalier Universitaire Vaudois (CHUV), Lausanne, Switzerland.

64. Department of Preventive Medicine, Division of Biostatistics, Keck School of Medicine, University of Southern California, Los Angeles, California, United States of America.

65. Laboratory of Epidemiology, Demography, and Biometry; Gerontology Research Center, National Institute on Aging, Bethesda, Maryland, United States of America.

66. Peninsula Medical School, Exeter, United Kingdom.

67. Department of Public Health, University of Helsinki, Helsinki, Finland.

68. Department of Medicine, Helsinki University Hospital, Helsinki, Finland and Folkhalsan Research Centre, Helsinki, Finland.

69. Twin Research & Genetic Epidemiology Department, King’s College London, St Thomas' Hospital Campus, London, United Kingdom.

70. Department of Genetics, University of North Carolina, Chapel Hill, North Carolina, United States of America.

71. Institute for Molecular Medicine Finland – FIMM, University of Helsinki, Helsinki, Finland.

72. Genome Technology Branch, National Human Genome Research Institute, Bethesda, Maryland, United States of America.

73. Institute of Medical Information Processing, Biometry, and Epidemiology, Ludwig‐Maximilians‐University München, München, Germany.

74. National Institute for Health Research, Oxford Biomedical Research Centre, University of Oxford, Headington, Oxford, United Kingdom.

75. Department of Genetics, Harvard Medical School, Boston, Massachusetts, United States of America.

76. A full list of members is provided in the Supplementary Note of Willer *Nature Genetics* 40:161‐169 (2008).

77. These authors contributed equally to this work.

78. Members of the writing team.

79. These authors jointly directed the project.

80. All authors are members of the Genetic Investigation of ANthropometric Traits (GIANT) Consortium.
